# CRISPR/Cas9 mediated *T7 RNA polymerase* gene knock-in in *E. coli* BW25113 makes T7 expression system work efficiently

**DOI:** 10.1186/s13036-021-00270-9

**Published:** 2021-08-12

**Authors:** Changchuan Ye, Xi Chen, Mengjie Yang, Xiangfang Zeng, Shiyan Qiao

**Affiliations:** 1 State Key Laboratory of Animal Nutrition, Ministry of Agriculture Feed Industry Centre, China Agricultural University, 100193 Beijing, China; 2 Beijing Key Laboratory of Bio-feed Additives, 100193 Beijing, China; 3 State Key Laboratory for Agro-Biotechnology, and Ministry of Agriculture and Rural Affairs, Key Laboratory for Pest Monitoring and Green Management, Department of Plant Pathology, China Agricultural University, 100193 Beijing, China; 4 National Feed Engineering Technology Research Centre, 100193 Beijing, China

**Keywords:** *E. coli*, CRISPR/cas9, T7 Expression System, Fluorescent Protein, 5-Aminolevulinic Acid, promoter variants

## Abstract

T7 Expression System is a common method of ensuring tight control and high-level induced expression. However, this system can only work in some bacterial strains in which the *T7 RNA Polymerase* gene resides in the chromosome. In this study, we successfully introduced a chromosomal copy of the *T7 RNA Polymerase* gene under control of the lacUV5 promoter into *Escherichia coli* BW25113. The T7 Expression System worked efficiently in this mutant strain named BW25113-T7. We demonstrated that this mutant strain could satisfactorily produce 5-Aminolevulinic Acid via C5 pathway. A final study was designed to enhance the controllability of T7 Expression System in this mutant strain by constructing a T7 Promoter Variants Library. These efforts advanced *E. coli* BW25113-T7 to be a practical host for future metabolic engineering efforts.

**Supplementary Information:**

The online version contains supplementary material available at 10.1186/s13036-021-00270-9.

## Introduction

Metabolic engineering plays a critical part in bio-based production of fuels, chemicals and materials from biomass, and often involves integration of multiple genes to re-direct metabolic fluxes [1]. In recent years, biosynthesis techniques have attracted increased attention. As synthetic biology applications have grown in complexity, so too has the sophistication of available genetic and biochemical tools. For example, researchers started to produce antibiotic using microbial fermentation in the mid-20th century [2]. Currently, a dramatic change in the scope and complexity manufacturing processes within the space of synthetic biology has occurred. Thanks to development of genetic engineering, accomplishments have progressed from simple reconstitution of biosynthetic pathways in heterologous hosts to ambitious refactoring efforts [3–5] that can produce high titers of medicinally relevant compounds (strictosamide, taxa-diene, etc. ) [6, 7] or amino acids (L-valine, L-threonine, etc. ) [8, 9]. Up to now, synthetic biologists have employed many tools to modify and reform bacterial strains to produce desired compounds. These tools include well-characterized sets of expression vectors [10], promoters for dynamic pathway control [11, 12], tunable protein degradation [13], and advanced methods for genome editing [14, 15].

Synthetic biologists prefer two organisms for expression and optimization of heterologous biosynthetic pathways: *Escherichia coli* and *Saccharomyces cerevisiae*. *E. coli* is the most widely used prokaryotic system that produces heterologous proteins for industrial production of bacterial metabolites by batch and fed-batch operations [16, 17]. *E. coli* is a gram-negative, facultative anaerobic and non-sporulating bacterium [18]. *E. coli* has been regarded as the workhorse of modern biotechnology in the microbial production of biofuels and biochemicals [19].

To express heterologous proteins in *E. coli*, researchers have designed and developed a large number of expression vectors based on specific signals (such as promoters) that bacteria can recognize. One of the most frequently used expression systems is the T7 expression system. T7 is a kind of bacteriophage with highly efficient self-replication after infection of *E. coli*. Early transcription is initiated by T7 RNA polymerase which can strongly initiate transcription of other genes [20]. The T7 expression system has several advantages such as: T7 RNA polymerase can recognize the T7 promoter specifically and initiate transcription of downstream genes; the mRNA transcribed by the T7 expression system is stable and has a strong translational signal [20, 21].

To ensure the T7 expression system works in *E. coli*, researchers have integrated genes that code *T7 RNA polymerase* into the genome of some *E. coli* strains such as BL21(DE3) [20, 21]. However, there are still some *E. coli* strains which have special characteristics, but lack the *T7 RNA polymerase* gene. Consequently, it is of great significance to find a convenient tool to integrate a large gene (such as *T7 RNA polymerase* gene, more than 4 kb) into the bacterial genome. A possible solution is the CRISPR/Cas9 system which has been developed for programmable and customizable genome engineering [22].

CRISPR/Cas is an RNA-guided system which enables site-specific induction of a double strand break (DSB) and programmable genome editing. CRISPR/Cas is an immune system in bacteria and archaea [23]. Because of modularization and easy handling, CRISPR/Cas9 was adopted to engineer genomes of *E. coli*, lactic acid bacteria, *streptomyces*, *Clostridium* and so on [24]. Among CRISPR/Cas9 methods, recombineering based on λ-Red has been widely employed for genome manipulation [1], which necessitates an editing template donor DNA and three phage λ proteins: Exo, Bet and Gam [25]. Gam prevents the host RecBCD nuclease (a kind of exonucleases in bacteria) from degrading the linear donor DNA, while Exo exonuclease resects the double-stranded DNA (dsDNA) and Bet binds the single-stranded DNA (ssDNA) to promote annealing of ssDNA to homologous chromosomal sequences [26]. Although there are many reports of successful insertion, deletion and replacement of prokaryotic genes using the CRISPR/Cas9 system, integration of large pieces of DNA into an *E. coli* chromosome is still facing many challenges, such as high off-target rates, difficulty in acquiring Donor DNA and finding a suitable insertion site [27]. In this study, we manage to integrate the *T7 RNA polymerase* gene into the chromosome of BW25113, which is a derivative of *E. coli* K-12 W1485. *Escherichia coli* BW25113 is a common laboratory strain that became the parent strain for the Keio collection, a major resource consisting of approximately 4,000 single-gene deletion mutants [28, 29]. The strain and its derivatives are being used in countless laboratories for a variety of studies, including systematic phenotypic surveys [30] and synthetic biology efforts [31–33]. Subsequently, we test the efficiency of the T7 Expression System in this mutant strain to ensure that heterogeneous expression of protein occurs.

**Fig. 1:**
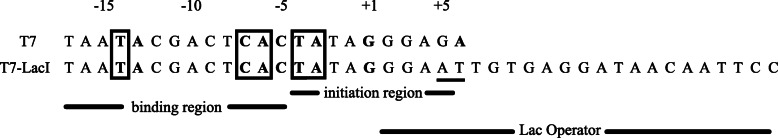
Comparison of phage original T7 and T7-LacI promoter sequences. The sequence of the non-template strand is shown about T7 and T7-Lac promoters. Positions inside the squares are conserved in all 47 natural phage promoter sequences. Positions in boldface type are common among all consensus promoters. Positions that are underlined are variant parts between original T7 and T7-Lac. The binding region (-17 to -5) and initiation region (-4 to + 6) of the T7 promoter are indicated

To make our findings more useful in metabolic engineering, we designed a library of T7-Lac promoter variants. Earlier studies indicated that the T7 promoter is composed of two functional domains: an upstream binding region from − 17 to -5 and an initiation region from − 4 to + 6. The promoter variants library was based on T7-Lac promoter [34], and the mutants were concentrated on the binding region (Fig. 1). We tested the expression strength of these promoter variants library in BW25113-T7, which will be useful in characterizing a number of aspects of promoter functions in metabolic engineering.

## Results

### Selection of integration site and design of Left and Right Arm

**Fig. 2:**
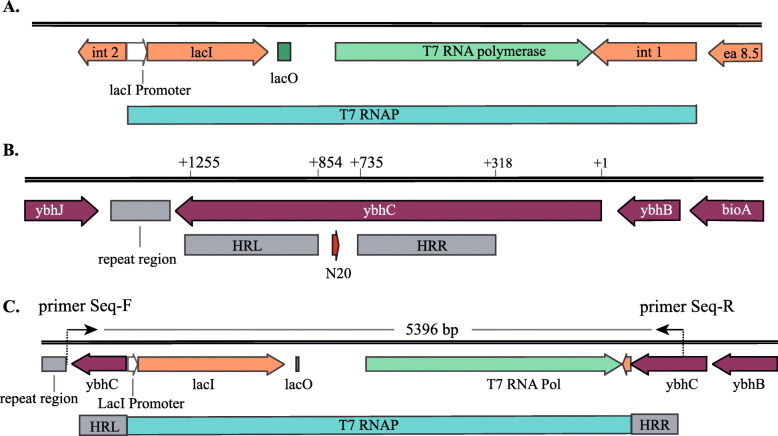
Gene map for targeted Cas9-mediated gene Knock-In. (**A**) Map of T7-RNAP in BL21(DE3), including *LacI*, *LacO* and *T7 RNA Polymerase* gene. (**B**) Integration site in BW25113. (**C**) map of T7-RNAP integration in ideal condition and the location of PCR product (5496 bp) for sequencing

*T7 RNA polymerase* gene, *LacI* gene, *LacI Operato*r and *Int 1* (T7-RNAP) were cloned from *E. coli* BL21(DE3). By referring to successful introduction in BL21(DE3), we decided to insert T7-RNAP into the *ybhC* gene site of BW25113. Besides, we verified that the *ybhC* gene only encodes an outer membrane lipoprotein, which would not negatively affect cell growth and metabolism after knocking out. The map of T7-RNAP in BL21(DE3) and the integration site in BW25113 are shown in Fig. 2 A and 2B. The left homologous arm (HRL) was set to be the downstream of the *ybhC* gene (854–1255 bp), and the right homologous arm (HRR) was set in the middle of the *ybhC* gene (318–735 bp). Both homologous arms were approximately 400 bp, which could have a high efficiency of recombination [35]. The map of T7-RNAP integration in BW25113 in ideal condition is shown in Fig. 2 C. All plasmids used in this research will be listed in Table 1.

**Table 1:** Strains and plasimds employed in this study

Strain or plasmid	Relevant characteristic(s)	Source and/or reference
Strain		
*Escherichia coli* DH5α	*F-,φ80dlacZ ΔM15,Δ(lacZYA -argF )U169, deoR , recA1 , endA1 ,hsdR17 (rK-, mK+), phoA, supE44 , λ-, thi-1, gyrA96 , relA1*	TransGen, Beijing
*Escherichia coli* BL21(DE3)	*fhuA2 [lon] ompT gal (λ DE3) [dcm] ΔhsdS λ DE3 = λ sBamHIo ΔEcoRI-B int::(lacI::PlacUV5::T7 gene) i21 Δnin5*	TransGen, Beijing
*Escherichia coli* BW25113	*F-, DE(araD-araB)567, lacZ4787(del)::rrnB-3, LAM-, rph-1, DE(rhaD-rhaB)568, hsdR514*	Laboratory stock
*Escherichia coli* BW25113-T7	BW25113 *int::(lacI::PlacUV5::T7 gene) ΔybhC*	This study
Plasmids		
pACYCD-Blank	*E. coli* cloning vector (p15a ori; CmR)	Laboratory stock
pACYCD-sYFP	pACYCD-Blank with *sYFP* gene, *LacI* gene and T7-LacI promoter	Laboratory stock
pCas	plasmid for CRISPR (temperature sensitive oriR101; KanR; the λ-Red operon under the control of arabinose-inducible promoter; *S. pyogenes*-derived cas9; sgRNA guided to ori-p15a under the control of lac operator)	Laboratory stock
pTarget	plasmid for CRISPR (p15a ori; CmR; sgRNA)	Laboratory stock
pTarget-ybhC	plasmid for CRISPR (p15a ori; CmR; sgRNA guided to *ybhC* gene)	This study
pACYCD-ybhC	pACYCD-Blank with *ybhC* gene (Fragment A)	This study
pACYCD-Donor DNA	pACYCD-Blank with Donor DNA	This study
pACYCD-sYFP-17A (and so on)	pACYCD-sYFP with T7 Promoter Mutation Library (Single-base substitution mutation)	This study
pET28b-ALA-LA	plasmid for biosyntheizing ALA (f1 ori; KanR; *hemA*; *hemL*; *LacI* gene and T7-LacI promoter)	Laboratory stock
pET28b-ALA-LAR	plasmid for biosyntheizing ALA (f1 ori; KanR; *hemA*; *hemL*; *RhtA*; *LacI* gene and T7-LacI promoter)	Laboratory stock

### Construction of plasmids for CRISPR and preparation of linear Donor DNA

Plasmid pCas prepared by our lab harbored the temperature sensitive oriR101 with repA101ts for curing at 37℃, kanamycin resistance gene (Kan^r^), and the λ-Red operon encoding Gam, Bet, and Exo proteins under the control of arabinose-inducible promoter *P*_*araB*_. This plasmid harbored *S. pyogenes*-derived *cas9* driven by endogenous promoters and small-guide RNA (sgRNA) guided to ori-p15a which is under the control of lac operator (Figure S1A).

Plasmid pTarget-ybhC was derived by targeting the pCRISPR crRNA region to the *E. coli* BW25113 *ybhC* gene, which harbored Chloramphenicol resistance (Chl^r^) and ori-p15a (Figure S1B).

Difficulty was acquiring the linear Donor DNA. The Donor DNA should contain the *T7 RNA Polymerase* gene and two homologous arms (HRL and HRR). The preparation of Donor DNA with high-fidelity was difficult as the *T7 RNA Polymerase* gene contains about 4.5 kb. In this research, we designed two intermediate cloning vectors for preparation of Donor DNA. We expected this approach would enhance efficiency of CRISPR/Cas9-mediated homologous recombination.

Primers used for preparation of Donor DNA are shown in Table 2. First, Fragment A (contained the *ybhC* gene, HRR, and HRL) was cloned from the *E. coli* BW25113 genome by Primer ybhC-F and ybhC-R. Then Fragment A was concatenated to pACYCD-Blank (p15a ori, Chl^r^) to assemble a new plasmid: pACYCD-ybhC (Figure S2A and S2B). Next, T7-RNAP (Fragment B) was cloned from the *E. coli* BL21(DE3) genome by Primer RNAP-F and RNAP-R and then Fragment B was assembled with pACYCD-ybhC. Subsequently, linear Donor DNA could be cloned from this new plasmid named pACYCD-Donor (Figure S2C and S2D). Donor DNA was purified, sequenced and confirmed to be without any mutations or absences.

**Table 2:** Primer pairs for CRISPR/Cas9 induced Knock-In

Primer	Sequences (5'to3')	Base Number	Note
Primer F1	**gcttgttggtacgc**ttctgccattcatccgcttattatcac	41	Positions in boldface type are homologous fragments designed for Takara In-Fusion
Primer R1	**accgcggcgtgggta**cctggcgttacccaacttaat	36	
Primer ybhC-F	tacccacgccgcggttattg	20	
Primer ybhC-R	aagcgtaccaacaagcgcca	20	
Primer F2	**cttttcgtgcgcgca**taacgttgttaatctgtacctggtc	40	Positions in boldface type are homologous fragments designed for Takara In-Fusion
Primer R2	**tggtgtccgggatctg**tatcgtttctggtcgcggcg	36	
Primer RNAP-F	cagatcccggacaccatcga	20	
Primer RNAP-R	atgcgcgcacgaaaagcatc	20	
Primer Donor-F	tacccacgccgcggttattg	20	
Primer Donor-R	aagcgtaccaacaagcgcca	20	
Primer N20-F	gttttagagctagaaatagcaagttaaaat	30	Embellished by 5’-phosphorylation
Primer N20-R	tctggaaacgaatcgtcagcactagtattatacctaggac	40	

### Confirmation of CRISPR/Cas9-Mediated Gene Knock-in in BW25113

*E. coli* lacks the Non-Homologous End Joining (NHEJ) mechanism to survive DNA cleavage [36] so the most transformants harboring pCas and pTarget would die due to co-expression of Cas9, tracrRNA and crRNA, and subsequent DSB at the protospacer. Upon DNA damage, bacteria initiate a coordinated SOS response that involves activation of RecA protein and expression of error prone polymerase V encoded by *umuDC* to facilitate mutagenic DNA repair and cell survival [37]. Together with the editing template Donor DNA and λ-Red proteins, the SOS response can repair the DSB and the donor DNA could be mediated into the chromosome by homologous recombination [24, 25, 38].

**Fig. 3:**
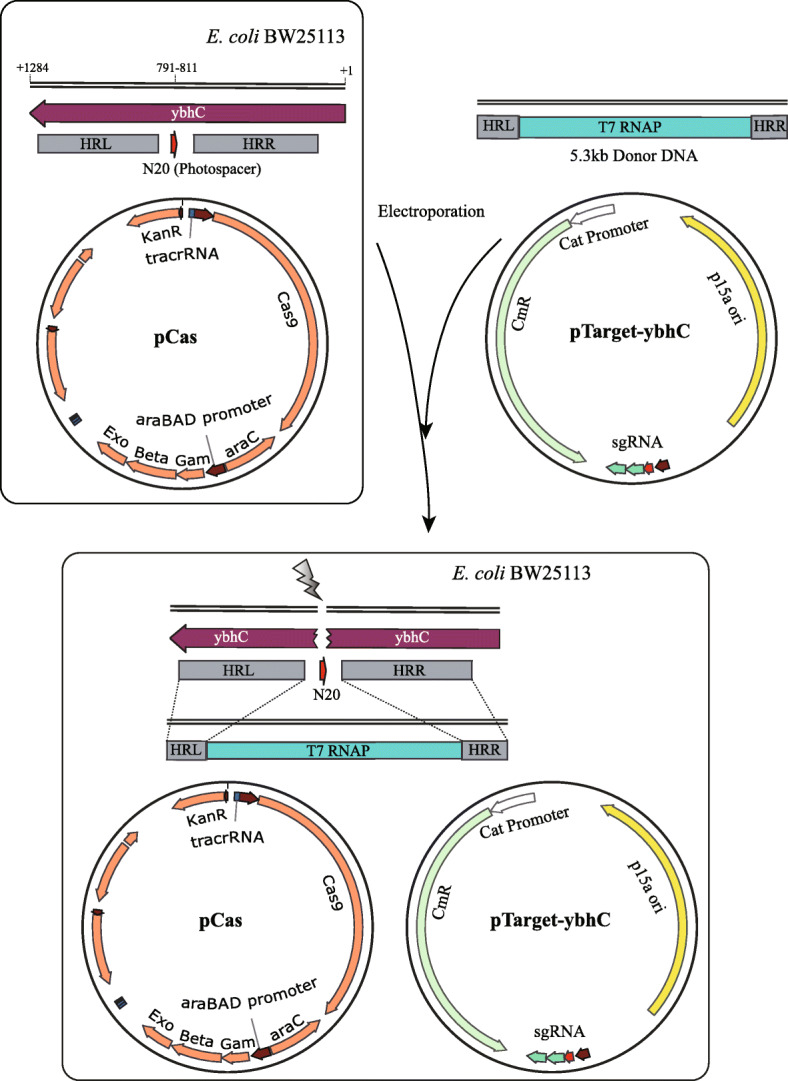
Schematic illustration of DSB induction and homologous recombination. After preparing competent cells, the pTarget-ybhC and Donor DNA which harbored T7-RNAP and homology arms (HRR and HRL) that targeted a chromosomal locus spanning the middle of ybhC gene and the DSB site were electroporated into cells

To make CRISPR/Cas9-mediated homologous recombination in BW25113, we electroporated pCas (encoding both Cas and λ-Red proteins) into *E. coli* BW25113, followed by Arabinose (Ara) induction of pCas-encoded λ-Red proteins Gam, Bet and Exo. After preparing competent cells, pTarget-ybhC and Donor DNA were co-electroporated into cells (Fig. 3). The plate of cells after co-electroporating is shown in Figure S3.

To attest the integration into target locus, six bacterial colonies were selected for colony PCR (Figure S4). Primers for the test were Primer RNAP-R and Test-F (Table 2 and Table S1).

Subsequently, a rigorous examination was conducted to estimate whether T7-RNAP was integrated into the genome. The whole T7-RNAP fragment and adjacent chromosomal region (5496 bp; Fig. 2 C) were cloned from this mutant strain by Primer Seq-F and Seq-R (Table S1). The PCR products were purified and sequenced to confirm that there were no mutations or absence in T7-RNAP. This mutant strain was named *E. coli* BW25113-T7.

### Growth characteristic of ***E. coli*** BW25113-T7

**Fig. 4:**
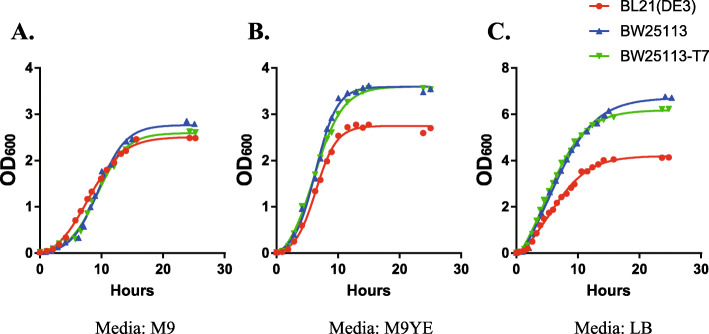
Comparison of the growth of ***E. coli*** strains BL21(DE3), BW25113 and BW25113-T7 in different medium. Cells were cultured in **(A)** M9 medium, **(B)** M9YE medium and **(C)** LB medium with 25 µg/mL Chloramphenicol. Bacteria contained pACYCD-Blank for Chloramphenicol resistance. Data are means of three replicates

Growth of *E. coli* BW25113-T7 in different medium was examined to assess whether CRISPR/Cas9-mediated T7-RNAP Knock-In affected the metabolic characteristics of the bacteria,

Growth of *E. coli* BW25113-T7 was compared with *E. coli* BW25113 and BL21(DE3). These three strains were cultured in standard M9 medium, special M9YE medium (standard M9 medium with additional 2 g/L Yeast Extract and 10 g/L Glucose) or standard LB medium (Fig. 4). There were no differences in growth rate among three strains in standard M9 medium (Fig. 4 A). However, growth rate of *E. coli* BW25113 and BW25113-T7 were higher than strain BL21(DE3) in nutrient medium such as standard LB medium or special M9YE medium (Fig. 4B C). Meanwhile, growth rate was similar between *E. coli* BW25113 and BW25113-T7 in these three types of medium (Fig. 4). These results indicated that induction of a chromosomal copy of the *T7 RNA Polymerase* gene does not impact growth characteristics of *E. coli* BW25113-T7.

### Efficiency of T7 expression system in BW25113-T7

**Fig. 5:**
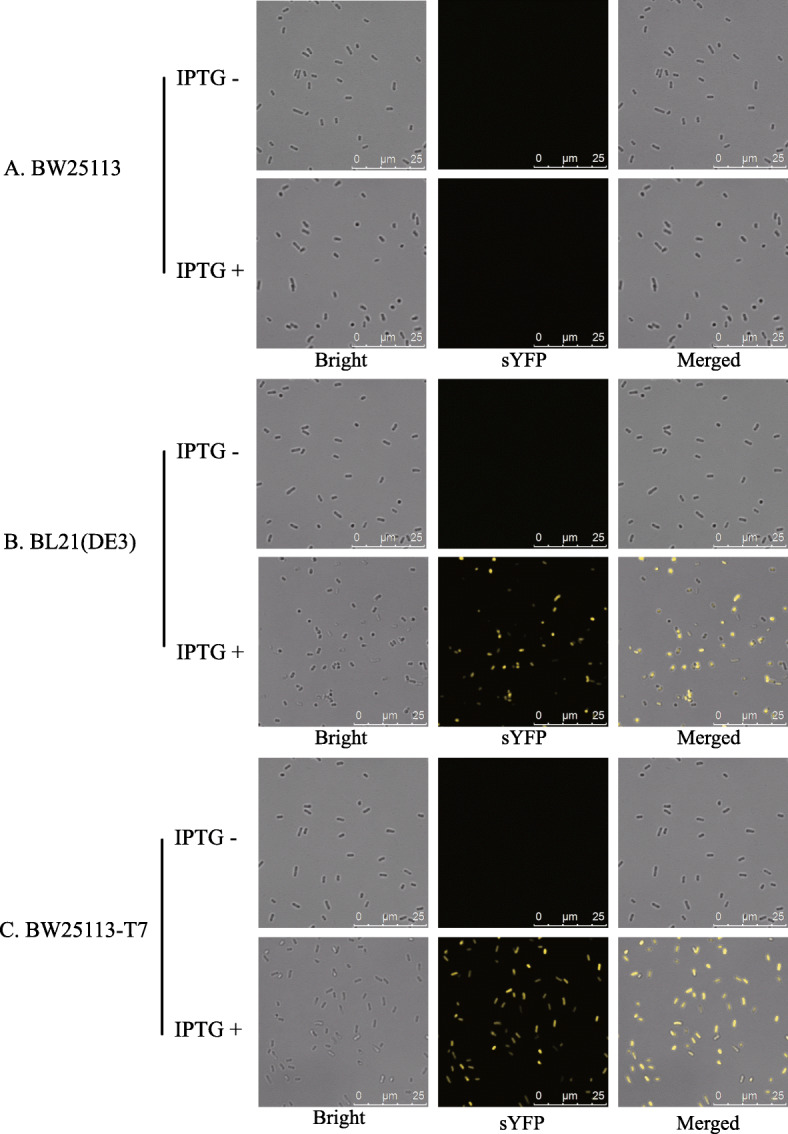
The T7 Expression system worked efficient in BW25113-T7 Expression of sYFP under control of T7-LacI promoter with (+) or without (-) IPTG induction in **(A)***E. coli* BW25113, **(B)***E. coli* BL21(DE3) and **(C)***E. coli* BW25113-T7

To explore whether the T7 expression system works in BW25113-T7, we detected fluorescence from the yellow fluorescent protein, sYFP, that is controlled by T7-Lac promoter (Figure S5) in BW25113-T7 with IPTG induction (Fig. 5). The T7-Lac promoter did not activate expression of sYFP in *E. coli* BW25113 because it lacked *T7 RNA Polymerase* gene (Fig. 5 A). After T7-RNAP knock-in, sYFP was expressed under control of T7-Lac promoter in *E. coli* BW25113-T7. Subsequently, the fluorescent signal could be macroscopically observed by Confocal Microscopy (Fig. 5B C). This result indicated that this mutant strain did express heterologous proteins efficiently through the T7 Expression System.

**Fig. 6:**
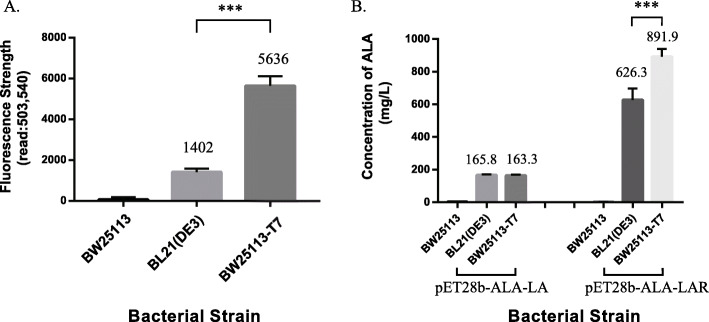
Confirmation of the efficiency of the T7 expression system in BW25113-T7. (**A**) Expression of sYFP under control of T7-LacI promoter in different bacterial strains. The fluorescent signal was detected by Multimode Reader (read: 503, 540) and was normalized to the cell density (OD_600_). (**B**) The biosynthesis of ALA (5-Aminolevulinic Acid) by T7 Expression System in different bacterial strains. These strains were cultured in Medium M9YE. All experiments were performed in triplicates. P values were calculated using Tukey’s multiple comparisons test (*P < 0.05, **P < 0.01, ***P < 0.001)

The quantitative fluorescent signal was detected by a Multifunctional Microplate Detector (Table 3). The fluorescent signal in *E. coli* BW25113-T7 was detected as 5636, which was about 4 times greater than that in *E. coli* BL21(DE3) (Fig. 6 A). It means that expression strength of sYFP under control of T7-Lac promoter in BW25113-T7 was greater than BL21(DE3) (p < 0.001). These results revealed that the T7 expression system has an enhanced efficiency in *E. coli* BW25113-T7.

**Table 3:** Expression of sYFP under control of T7-LacI promoter in different bacterial strains

Strains	Plasmid	Expressed genes	Fluorescent signal
BW25113	pACYCD-sYFP	sYFP	63 ± 89
BW25113-T7	pACYCD-sYFP	sYFP	1402 ± 221
BL21(DE3)	pACYCD-sYFP	sYFP	5635 ± 43

The results are the average of three individual experiments

### Production of 5-Aminolevulinic Acid via C5 pathway

To further ascertain the biosynthetic efficiency and metabolic engineering potential in *E. coli* BW25113-T7, we designed a process for 5-Aminolevulinic Acid biosynthesis in this mutant strain.

**Fig. 7:**
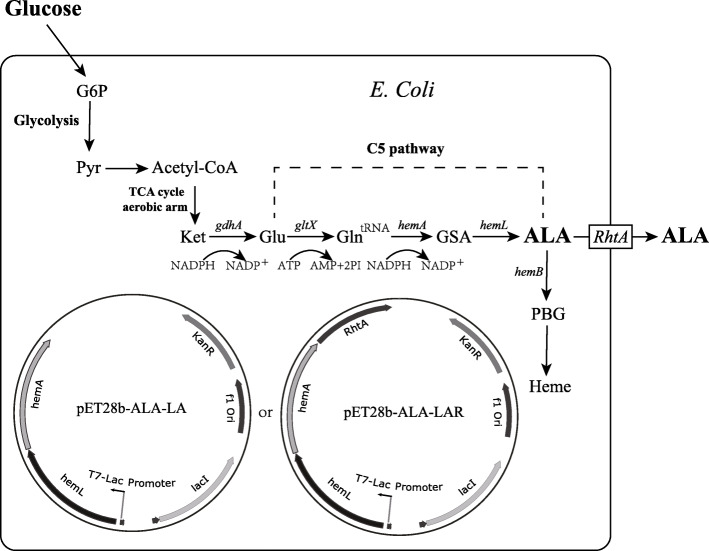
Schematic presentation of ALA production from glucose in E. coli via C5 pathway. G6P, glucose-6-phosphate; Pyr, pyruvate; Ket, α-ketoglutarate; Glu, glutamate; Gln^tRNA^, Glutamyl-tRNA; GSA, glutamate 1-semialdehyde aminotransferase; ALA, 5-aminolevulinic acid; PBG, porphobilinogen

5-Aminolevulinic Acid (ALA), a five-carbon amino acid, is a key intermediate involved in the biosynthesis of tetrapyrrole [39]. ALA recently received much attention due to its potential applications in many fields, such as tumor-localization and photodynamic therapy for various cancers [40–42]. ALA is also used as a selective biodegradable herbicide and insecticide in agricultural applications due to its nontoxicity to crops, animals, and humans [43, 44]. Currently, biosynthesis of ALA has become the focus of much research [45]. In living organisms, there are two major pathways described for ALA biosynthesis: C4 pathway and C5 pathway [43]. The C5 pathway occurs in higher plants, algae, and many bacteria including *E. coli* [46]. In the C5 pathway, glutamate is the only substrate for biosynthesis of ALA. In *E. coli*, a metabolic strategy of ALA biosynthesis via C5 pathway has been developed [39]. Through this strategy, ALA can be synthesized in *E. coli* by over-expressing the key genes, *hemA* and *hemL*. In this research, we used a similar process for ALA biosynthesis (Fig. 7). In our mutant strain, the key genes, *hemA* and *hemL*, were expressed efficiently and controlled by T7 Expression System.

Synthesis efficiency of ALA was compared among three strains of *E. coli*. After transferring pET28b-ALA-LA or pET28b-ALA-LAR (Fig. 7), cells were cultured in special M9YE medium. Genes, *hemA* and *hemL*, were over-expressed under control of T7-Lac promoter in expression vector pET28b-ALA-LA, while an ALA exporter gene *RhtA* was over-expressed additionally in expression vector pET28b-ALA-LAR. *RhtA* is classified as amino acid secondary transporters and capable of translocating a variety of amino acids and related compounds, such as dipeptide and amino acid analogs. As ALA has similar chemical structure to glycylglycine and its physical properties are close to native amino acids carrying uncharged side chains, amino acid exporters with wide substrate specificity such as *RhtA* was supposed to play roles in ALA export. *RhtA* encodes an inner membrane transporter, which is responsible for threonine and homoserine efflux transport.

After 24 h of fermentation, ALA concentration was determined (Table 4; Fig. 6B). Concentration of ALA in the fermentation broth of BW25113-T7 was 165.8 mg/L, which was similar to that of BL21(DE3) when *hemL* and *hemA* were over-expressed. However, both of these two bacterial strains had a higher production of ALA when *RhtA* was also over-expressed. After 24 h cultivation, a titer of ALA (891.9 mg/L) was achieved in BW25113-T7, which was significantly higher than that of BL21(DE3) (626.3 mg/L). Notably, efficiency of ALA metabolism synthesis in BW25113-T7 was significantly higher (p < 0.001) than that of BL21(DE3) when these three key genes (*hemA*, *hemL* and *RhtA*) were over-expressed simultaneously by the T7 Expression System.

**Table 4:** ALA production in different bacterial strains expressing various related genes

Strains	Plasmid	Expressed genes	ALA accumulation (mg/L)
BW25113	pET28b-ALA-LA	*hemA*, *hemL*	2.9 ± 0.9
BW25113-T7	pET28b-ALA-LA	*hemA*, *hemL*	165.8 ± 2.7
BL21(DE3)	pET28b-ALA-LA	*hemA*, *hemL*	163.3 ± 2.6
BW25113	pET28b-ALA-LAR	*hemA*, *hemL*, *RhtA*	2.4 ± 0.3
BW25113-T7	pET28b-ALA-LAR	*hemA*, *hemL*, *RhtA*	626.3 ± 32.8
BL21(DE3)	pET28b-ALA-LAR	*hemA*, *hemL*, *RhtA*	891.3 ± 22.3

The results are the average of three individual experiments

### Construction of the T7 Promoter Variants Library

The T7 Expression system was driven by T7 promoter which is recognized by *T7 RNA polymerase*. Relatively small amounts of bacteriophage *T7 RNA polymerase* can direct most of the resources of an *E. coli* cell toward expression of a specific target gene [20]. However, such active transcription can be a problem in expression systems where *T7 RNA polymerase* is supplied to the cell by induction. In this research, we constructed a library of T7-Lac promoter variants to make the T7 Expression System in *E. coli* BW25113-T7 more controllable.

This Promoter Variants Library was based on T7-Lac promoter, and the mutation sites were concentrated on the binding region (Fig. 1). Desired mutations were introduced into the test promoter (Px) in pACYCD-sYFP by Inverse-PCR and mismatched primers as described in Table S2. To improve the efficiency of rephrase-self connection recombination, the reverse primer was embellished by 5’-phosphorylation.

**Fig. 8:**
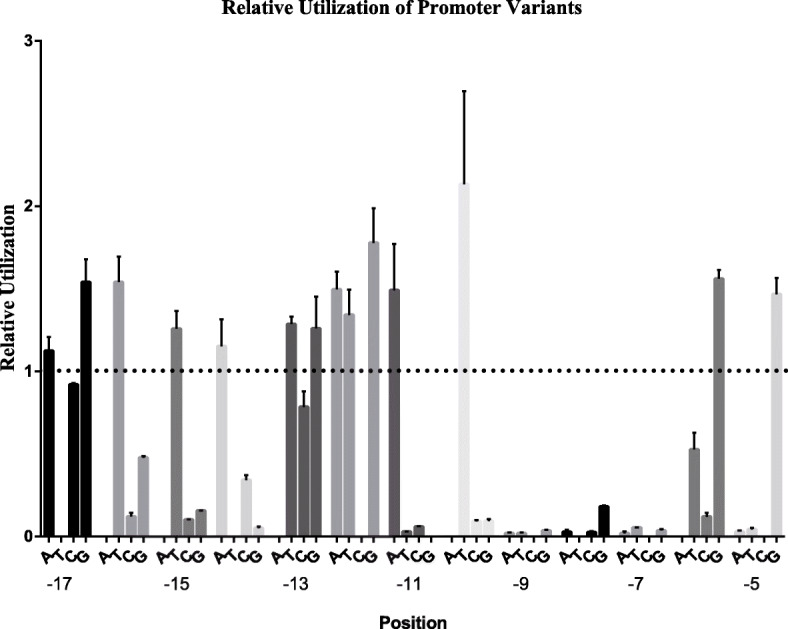
Characterization of T7 RNAP promoter variants: Relative activity of promoter variants. Promoter variants were identified by referring to the base in the non-template strand of the DNA (e.g., a -17 A promoter). The height of each bar indicates the activity of each promoter relative to that of the consensus promoter (data are from Table 5). All experiments were performed in triplicates

The resulting plasmid contains the test promoter recognized by T7 RNAP and sYFP which is driven by this promoter (Figure S5). Utilization of each of these promoters may be determined by comparing production of a runoff product (Fluorescent signal) of characteristic size from each promoter (Fig. 8). The results are summarized in Table 5. For convenience, we identify promoter variants by referring to the base in the non-template strand of the DNA (e.g., a -17 A promoter).

**Table 5:** Utilization of T7 promoter variants

Plasmid^*a*^	Px^*b*^	Consensus base^*c*^	Naturally occurring promoters^*d*^	Relative promoter strength^*e*^	Standard error
pACYCD-sYFP	consensus			1	
pACYCD-sYFP-17A	-17A	T		1.12	0.07
pACYCD-sYFP-17C	-17C			0.92	0.01
pACYCD-sYFP-17G	-17G		*φ*1.1A, 4C, 4.7	1.54	0.11
pACYCD-sYFP-16C	-16C	A		0.12	0.02
pACYCD-sYFP-16G	-16G			0.48	0.01
pACYCD-sYFP-16T	-16T		*φ* 4.7	1.54	0.13
pACYCD-sYFP-15C	-15C	A		0.10	0.00
pACYCD-sYFP-15G	-15G			0.16	0.00
pACYCD-sYFP-15T	-15T			1.26	0.09
pACYCD-sYFP-14A	-14A	T		1.15	0.13
pACYCD-sYFP-14C	-14C			0.34	0.02
pACYCD-sYFP-14G	-14G			0.05	0.01
pACYCD-sYFP-13C	-13C	A	*φ*4c	0.78	0.08
pACYCD-sYFP-13G	-13G			1.26	0.16
pACYCD-sYFP-13T	-13T		*φ* 3.8, 4.7	1.29	0.04
pACYCD-sYFP-12A	-12A	C		1.50	0.09
pACYCD-sYFP-12G	-12G		*φ* 3.8	1.78	0.17
pACYCD-sYFP-12T	-12T			1.34	0.13
pACYCD-sYFP-11A	-11A	G	*φ* OL, 3.8	1.49	0.23
pACYCD-sYFP-11C	-11C			0.06	0.00
pACYCD-sYFP-11T	-11T			0.03	0.00
pACYCD-sYFP-10C	-10C	A		0.09	0.01
pACYCD-sYFP-10G	-10G			0.09	0.01
pACYCD-sYFP-10T	-10T			2.13	0.46
pACYCD-sYFP-9A	-9A	C		0.02	0.00
pACYCD-sYFP-9G	-9G			0.04	0.00
pACYCD-sYFP-9T	-9T			0.02	0.00
pACYCD-sYFP-8A	-8A	T		0.03	0.01
pACYCD-sYFP-8C	-8C			0.03	0.01
pACYCD-sYFP-8G	-8G			0.18	0.01
pACYCD-sYFP-7A	-7A	C		0.02	0.01
pACYCD-sYFP-7G	-7G			0.04	0.01
pACYCD-sYFP-7T	-7T			0.05	0.00
pACYCD-sYFP-6C	-6C	A		0.12	0.02
pACYCD-sYFP-6G	-6G			1.56	0.04
pACYCD-sYFP-6T	-6T			0.53	0.08
pACYCD-sYFP-5A	-5A	C		0.03	0.01
pACYCD-sYFP-5G	-5G			1.47	0.08
pACYCD-sYFP-5T	-5T			0.04	0.01

^a^Plasmids in the pACYCD series were constructed in this work

^b^Promoter variants are designated as P-nX, where n indicates the position in the promoter and X indicates the base in the non-template strand

^c^The base in the non-template strand of the consensus promoter at the indicated position

^d^Naturally occurring T7 RNA Polymerase class II promoters that contain the indicated substitution; all class II promoters have two or more substitutions

^e^Plasmid templates were transcribed as described in Fig. 11, and the products (Fluorescent signal) were measured by Synergy™ HTX Multifunctional Microplate Detector

The utilization of each mutant promoter was then expressed relative to that of the consensus promoter when it was present at the position of Px

The Promoter Variants Library constructed in this study was not for the mechanism of the intensity change caused by single base mutation, but to apply this Library in biosynthetic engineering. By construction of this Library, promoter variants with different strengths were obtained. Therefore, expression of targeted gene can be regulated conveniently by replacing T7-Lac promoter variants with the required strength (Table 5). For convenient applying in synthetic biology, the re-ordered Promoter Variants Library (from the strongest promoter to the weakest promoter) was listed in the supplemental material (Figure S6 and Table S3). Construction of the Promoter Variants Library makes the T7 expression system more controllable in our newly engineered-strain: BW25113-T7.

## Discussion

As a common strategy of high-level induced expression, T7 expression system can ensure tight control to make the gene to be expressed in a plasmid under the control of a T7 phage promoter. *T7 RNA polymerase* is highly selective for its own promoters, which do not occur naturally in *E. coli*. A relatively small amount of *T7 RNA polymerase* provided from a cloned copy of T7 gene is sufficient to direct high-level transcription from a T7 promoter in a multicopy plasmid [20]. Specific mRNAs produced by *T7 RNA polymerase* are relatively stable and can rapidly saturate the translational machinery of *E. coli*, so that the rate of protein synthesis from the mRNA will depend primarily on efficiency of its translation. When mRNA is efficiently translated, a target protein can accumulate to greater than 50 % of the total cell protein in three hours or less [20, 21].

To express heterologous proteins by the T7 Expression System, active *T7 RNA polymerase* was delivered to bacterial strains such as BL21(DE3) by induction of a chromosomal copy of *T7 RNA Polymerase* gene under control of the lacUV5 promoter (Fig. 2 A) [20, 21]. Expression plasmid vectors have been constructed to allow target genes to be placed under control of the T7-Lac promoter and to be expressed in bacteria strains which carry an inducible gene for *T7 RNA Polymerase* [34]. Transcription can be repressed strongly by *lac* repressor bound to an operator centered 15 base-pairs downstream from the RNA start. But, *T7 RNA polymerase* initiates transcription very actively from this T7-Lac promoter-operator combination in the absence of a repressor, or in the presence of a repressor plus inducer. To achieve a low basal expression of target genes, expression plasmid vectors usually carry a *LacI* gene that provides enough *lac* repressor to repress both the T7-Lac promoter in the multicopy vectors and the chromosomal gene for *T7 RNA polymerase*, which is controlled by the lacUV5 promoter. Upon induction, the usual high levels of expression are obtained [34, 47].

However, only strains with the *T7 RNA Polymerase* gene can use the T7 Expression System for efficient and controllable protein expression. Some strains commonly used in metabolic engineering (such as *E. coli* BW25113) cannot utilize the T7 Expression System even though they may have some superior characteristics (a higher growth rate or lower level of enterotoxin) [48, 49]. In this study, the CRISPR/Cas9 system combined with the λ-Red homologous recombination repair system was used to complete the insertion of the *T7 RNA Polymerase* gene into the genome of *E. coli* BW25113. We found that induction of a chromosomal copy of the *T7 RNA Polymerase* gene did not affect growth characteristics and enabled the T7 Expression System in this mutant strain for heterologous protein expression or gene over-expression.

The CRISPR/Cas9 system is an efficient and highly targeted gene editing tool. Since its discovery, Cas9 has been extensively used for genome editing in multiple organisms [27]. Although the full potential of CRISPR/Cas9 has not yet been harnessed, this technology has brought forth revolutionary changes in genomic research, including genome editing, regulation, and imaging [24, 50]. Despite Cas9’s great potential for both research and therapeutics, improvements can still be made in its specificity, efficiency, and spatiotemporal control [51]. There are still some challenges of CRISPR/Cas9 mediated large DNA Knock-In, such as high off-target rate, difficult access for Donor DNA, and high mutation rate of knock-in fragments [24, 52]. Based on the principle of ensuring fidelity of the repair template (Donor DNA), this study constructed two intermediate plasmids (pACYCD-ybhC and pACYCD-Donor) to acquire Donor DNA with high fidelity. The large DNA (T7-RNAP, 5.4 kb) was inserted into the genome of *E. coli* BW25113, and this mutant strain was named BW25113-T7.

The working efficiency of the T7 expression system in *E. coli* BW25113-T7 was confirmed by Fluorescent-Protein-Based Reporter Gene System (Fig. 5). In this mutant strain, the T7 expression system expressed the sYFP fluorescent protein and the efficiency of protein expression was higher than that of BL21 (DE3) (Fig. 6 A). Therefore, *E. coli* BW25113-T7 might provide the ability to produce high titers of simple peptides by the T7 Expression System.

The advantage of this protein expression could also extend to metabolic engineering. We speculate that the T7 Expression System in *E. coli* BW25113-T7 has greater efficiency of expression efficiency than BL21(DE3). Thus, three genes overexpressed by T7 Expression System (*hemA*, *hemL* and *RhtA*) in BW25113-T7 were all expressed at higher levels than BL21(DE3). When key synthetase genes, *hemA* and *hemL*, were over-expressed by the T7 Expression System, there were no differences between BW25113-T7 and BL21 (DE3) in ALA production (Fig. 6B). This result is easily explained, as the accumulation of compounds is more closely related to the activity and quality than quantity of synthetase in metabolic engineering [10, 39, 53]. However, when the exporter gene, *RhtA*, was over-expressed in addition to *hemA* and *hemL*, production of ALA in BW25113-T7 was significantly higher than that of BL21(DE3) (Fig. 6B). Therefore, we speculate that rate of ALA export in the BW25113-T7 strain is higher than that of BL21 (DE3) due to greater expression of *RhtA*. As a result of accelerated ALA export, *E. coli* BW25113-T7 accumulated 42.4 % more than *E. coli* BL21(DE3) (Fig. 6B).

Our mutant strain BW25113-T7 retains the characteristic rapid growth of the original strain. Although there were no differences of growth rate between BW25113-T7 and BL21(DE3) in minimal medium, BW25113-T7 showed faster growth when cultured in nutrient medium (Fig. 4). These results demonstrated that after T7-RNAP Knock-In, this mutant strain has certain advantages in metabolic engineering and synthetic biology. Otherwise, *E. coli* BW25113-T7 still has potential for genetic modification. This strain can be further edited and modified according to specific metabolic engineering strategies to make it more suitable for production of required compounds.

There are some disadvantages with the T7 Expression System. Due to the abnormally high activity of T7 polymerase, this inducible expression system is not the optimal choice for many applications in metabolic engineering. When the T7 Expression System was activated upon induction, a target protein can accumulate to greater than 50 % of the total cell protein [20]. The high levels of protein expression would be a burden on cells, which leads to a negative impact on bacterial growth. Besides, synthetic biologists working in metabolic engineering are concentrated on activity and quality of synthetase not just quantity of synthetase [10, 39, 53]. Therefore, application of T7 Expression System still has some limitations in metabolic engineering. As an attempt to enhance controllability and practicability of the T7 Expression System in this mutant strain BW25113-T7 for metabolic engineering, we designed a series of experiments to construct a T7 Promoter Variants Library.

The essence of this T7 Promoter Variants Library is a series of promoter variants obtained by single-base mutation of each of the 13 bases on the Binding Region of the T7-Lac promoter (Fig. 1). Characterization of this series of promoters was measured by Fluorescent-Protein-Based Reporter Gene System (Fig. 8; Table 5). Based on such a library of promoters, expression of targeted proteins can be adjusted in a controlled manner such that application of the T7 expression system can be extended. We can flexibly regulate expression of targeted proteins according to specific needs to obtain the best yield of metabolites. For example, when we desired a higher intensity expression of heterologous protein, the T7-Lac promoter can be replaced as a variant with a higher expression intensity.

In conclusion, the *T7 RNA Polymerase* gene was inserted into *E. coli* BW25113 genome by CRISPR/Cas9 to construct a new *E. coli* strain in this study. This mutant strain maintained the advantages the original strain (such as the rapid growth rate) and allowed for efficient and controllable protein expression using the T7 Expression System. Further study of constructing T7 Promoter Variants Library enhanced the application of this strain in synthetic biology and provided a technical basis for industrial production in the future. Accordingly, these efforts advanced *E. coli* BW25113-T7 to be a practical host for future metabolic engineering efforts.

## Materials and methods

### ***E. coli*** Strains

DH5α (TransGen, Beijing) was used for cloning plasmid. BL21(DE3) (TransGen, Beijing) was used for cloning *T7 RNA polymerase* genes, *LacI* and *LacO* (T7-RNAP), from its chromosomal genome. BW25113 strains were used for CRISPR/Cas9-induced DSB and recombination. All *E. coli* strains were routinely cultured in standard LB medium when not mentioned otherwise.

### Selection of integration site and design of homologous recombination

Sequence of T7-RNAP in BL21(DE3) genome and the integration site of BW25113 were both confirmed in NCBI. The function and detailed message of the *ybhC* gene was verified in NCBI and BioCyc Database. As the recognition site for sgRNA, N20 site directs the Cas9 protein to enable site-specific induction of a DSB. The N20 site was found by BROAD international design tool, which is available at: http://www.broadinstitute.org/rnai/public/analysis-tools/sgrna-design.

### Plasmids Construction for CRISPR and preparation of linear Donor dsDNA by PCR

Table 2 contains all primer pairs we designed for gene cloning and intermediate plasmid construction. Plasmid pCas (Figure S1A) and pTarget were prepared in our laboratory. Plasmid pTarget-ybhC was constructed by Inverse-PCR and T4-Ligation (T4 DNA Ligase, NEB, England) to replace the N20 fragment (Figure S1B) with specific primers (Table 2). Plasmid pACYCD-ybhC and pACYCD-Donor were both constructed for preparing Donor DNA (Figure S2) by In-Fusion® HD Cloning Kit (Takara, Japan). Donor DNA was cloned from pACYCD-Donor by Hi-Fi PCR (Phusion® High-Fidelity PCR Master Mix, NEB, England).

All plasmids used in this research will be listed in Table 1.

### Electroporation, Cell Recovery, and Plating

For transformation, the plasmid or linear DNA were electroporated into competent cells in the pre-chilled cuvette (0.1 cm) using Bio-Rad MicroPulser (1.8 kV, time constant > 5.0 ms). For selection, 25 µg/mL chloramphenicol (Chl) or 50 µg/mL kanamycin (Kan) were used alone or in combination. For induction of λ-Red proteins and lac operator, 1 mM arabinose and 1 mM IPTG were used.

To prepare cells harboring pCas, cells cultured at 37℃ (OD_600_ = 0.45–0.55) were made competent, mixed with pCas (100 ng) and subjected to electroporation, after which the cells were recovered in SOC medium (1 mL) for 1 h at 30℃, plated onto the Kan plate, and cultured at 30 ℃ for 18–24 h.

For CRISPR/Cas9-mediated homologous recombination, cells harboring pCas were cultured at 30℃ in medium containing Kan and Arabinose and made competent. After co-electroporation of Donor DNA (400 ng) with pTarget-ybhC (100 ng), cells were recovered in SOC (1 mL) medium for 1 h at 30℃, plated onto Chl/Kan plate, and cultured at 30℃ for 18–24 h.

For elimination of pTarget-ybhC, cells harboring both pCas and pTarget were cultured at 30℃ in medium containing Kan and IPTG for 2 h. Cells were plated onto Kan plates and cultured at 30℃ for 18–24 h.

For elimination of pCas, cells harboring pCas were cultured at 37℃ in the medium without any antibiotic for 12–16 h. Then the cells were plated onto non-antibiotic plates and cultured at 37℃ for 12–16 h.

### Verification of Gene Integration by Colony PCR and DNA-seq

To verify correct integration of T7-RNAP, we designed Primer Seq-F and Primer Seq-R as shown in Table S1. Primer Seq-F targeted downstream of the *ybhC* gene (1267–1288 bp), while Primer Seq-R targeted upstream of the *ybhC* gene (261–282 bp). If integration was successful, colony PCR of the recombinants would yield a ≈ 5 kb amplicon, which contains HRL, T7-RNAP and HRR. Absences or mutations of the integration fragment were verified by DNA-sequencing.

All DNA-sequencing for this research was conducted by Beijing Ruibio BioTech Co., Ltd (China, Beijing).

### Growth of bacteria in different medium

All *E. coli* strains were grown at 37℃ in conical flask (250 mL) containing M9 medium (100 mL), M9YE medium or LB medium with chloramphenicol (25 µg/mL). Growth was measured by monitoring optical density at 600 nm (OD_600_) using a spectrophotometer.

### Confirmation of T7 Expression System in BW25113-T7 by sYFP Reporter Gene System

Efficiency of the T7 expression system was confirmed by a fluorescent protein reporter assay system. The plasmid was named as pACYCD-sYFP, which harbored ori-p15a, Chloramphenicol resistance (Chl^r^), LacI gene, T7-Lac promoter [34] and sYFP gene (Figure S5). This fluorescent protein could reflect 540 nm light by the 503 nm exciting light. This optical signal could be observed microscopically by Confocal Microscopy and accurately detected by a Fluorescence Detector. This plasmid was transformed into *E. coli* BL21(DE3) (as Positive Control), *E. coli* BW25113 (as Negative Control) and *E. coli* BW25113-T7.

### Confocal Microscopy

Cells harboring pACYCD-sYFP were cultured at 37℃ for 2 h. Then IPTG with a final concentration of 1 mM was added to the induced group. The bacterium solution was observed under Confocal Microscopy after 4 h of induction.

All strains were prepared for Confocal Microscopy as stated above for fluorescence measurements and prepared as a wet mount. Confocal microscopy was performed using a Leica TCS SP2 inverted confocal microscope (Leica Microsystems, Wetzlar, Germany) equipped with a 63× water-corrected objective in multitrack mode. Collected images were analyzed with the Leica Application Suite for Advanced Fluorescence (LAS AF, version 2.35) software (Leica Microsystem).

### Fluorescence intensity measured by Multifunctional Microplate Detector

Cells harboring pACYCD-sYFP were cultured in LB containing Chl at 37℃ for 2 h. Then IPTG with a final concentration of 1 mM was added to the induced group. The fermented liquid was added to a 96 well plate after 4 h of induction, then accurate fluorescent data was detected by Synergy™ HTX Multifunctional Microplate Detector (BioTek Instruments, America). The excitation light was set as 503 nm and the receiving light was set as 540 nm. Collected data was analyzed with the Gen5™ V2 Data Analysis Software (BioTek Instruments, Inc.).

### Analytical procedures of ALA production

Cells harboring pET28b-ALA-LA or pET28b-ALA-LAR were cultured in M9YE containing Kan at 37℃ for 2 h. Then IPTG with a final concentration of 0.1 mM was added to the induced group. To analyze ALA production, culture (30 mL) after induction for 24 h was centrifuged (12,000 g for 2 min at 4℃). The supernatant was used for extracellular ALA analysis. ALA concentration was analyzed using modified Ehrlich’s reagent [54]. Specifically, 2 ml of standard or sample (after diluted) was mixed with 1 ml 1.0 M sodium acetate (pH 4.6) in a cuvette, and 0.5 ml acetylacetone (2,4-pentanedi-one) was added to each cuvette. Then the mixtures were heated at 100 ℃ for 15 min. After cooling for 15 min, 1 ml of the reaction mixture and 1 ml freshly prepared modified Ehrlich’s reagent were mixed together. After 30 min, the absorbance at 554 nm was measured. Standard plot for ALA measurement is shown in Figure S7.

### Construction of the T7 Promoter Variants Library

Plasmid pTarget-ybhC was constructed by inverse PCR and T4-Ligation (T4 DNA Ligase, NEB, England) to make the single-base substitution mutation (Figure S5). The primers used are listed in Table S2. The plasmids were all sequenced to ensure that the single-base substitution mutation was introduced.

### Data Analysis

Data for fluorescence intensity were subjected to analysis of variance (ANOVA) by GraphPad Prism (version 7.00). The quantitative fluorescent data was normalized to the cell density (OD_600_). Error bars indicate standard error of the mean (SEM). P values were calculated using Tukey’s multiple comparisons test (*P < 0.05, **P < 0.01, ***P < 0.001).

## Supplementary Information

**Additional file 1: Figure S1.** Map of plasmids which were constructed for CRISPR. A) Map of pCas, which harbored the temperature sensitive oriR101 with repA101ts, kanamycin resistance gene, the λ-Red operon encoding Gam, Bet, and Exo proteins under the control of arabinose-inducible promoter ParaB,* S. pyogenes*-derived cas9 driven by endogenous promoters and sgRNA guided to ori-p15a which is under the control of lac operator. **B)** Map of pTarget-ybhC, which harbored Chloramphenicol resistance, ori-p15a and sgRNA guided to *E. coli* BW25113 ybhC gene.

**Additional file 2: Figure S2.** Construct of intermediate cloning vectors for preparing Donor DNA. A) Fragment A cloned from BW25113 was concatenated to pACYCD-Blank to assemble pACYCD-ybhC. B) Fragment B cloned from BL21(DE3) was concatenated to pACYCD-ybhC to assemble pACYCD-Donor. C) Map of pACYCD-Donor. D) The map of Donor DNA, which contains HRL, T7-RNAP and HRR.

**Additional file 3: Figure S3.** Confirmation of CRISPR/Cas9-mediated DSB in E. coli BW25113. The macroscopic images of colony formation after co-electroporation of Donor DNA and pTarget-ybhC.

**Additional file 4: Figure S4.**Detection of successful RNAP Knock in clones of BW25113-T7. A) The location of colony PCR product in BW25113-T7 genome. B) Lane M, 1kb Plus DNA ladder (TransGen, Beijing); lanes 1 was blank; lanes 2 to 8 were samples of colony PCR; lanes 8 and 9, positive control and negative control, respectively.

**Additional file 5: Figure S5.**Map of pACYCD-sYFP. A) Sequence of T7-LacI promoter. B) Map of T7-LacI promoter. C) Map of pACYCD-sYFP (p15a ori; CmR; lacI; Lac operator and T7 promoter; sYFP).

**Additional file 6: Figure S6.** Characterization of T7 RNAP promoter variants: Relative activity of promoter variants (From Strongest to Weakest). Promoter variants were identified by referring to the base in the non-template strand of the DNA (e.g., a -17A promoter). The height of each bar indicates the activity of each promoter relative to that of the consensus promoter (data are from Table 5). All experiments were performed in triplicates.

**Additional file 7: Figure S7.**Standard Plot for ALA measurement.x: the absorbance at 554 nm; y: ALA concentration of sample after diluted (mg/L)

**Additional file 8.**

**Additional file 9.**

**Additional file 10.**

## Data Availability

The majority of data generated or analyzed during this study are included in this published article or in the supplementary information. The data not shown in the manuscript are available upon request from the corresponding author.

## Abbreviations

*E. coli*: *Escherichia coli*
DSB: Double strand break
dsDNA: Double-stranded DNA
ssDNA: Single-stranded DNA
T7-RNAP: *T7 RNA polymerase* gene
*LacI* gene: *LacI Operator* and *Int 1*
HRL: Left homologous arm
HRR: Right homologous arm
Kan^r^: Kanamycin resistance gene
sgRNA: Small-guide RNA
Chl^r^: Chloramphenicol resistance
NHEJ: Non-Homologous End Joining
ALA: 5-Aminolevulinic Acid
