# Supplementary material for: CRISPR/Cas9 mediated T7 RNA polymerase gene knock-in in E. coli BW25113 makes T7 expression system work efficiently
Source: J Biol Eng. 2021 Aug 12;15:22. doi: 10.1186/s13036-021-00270-9 (PMC8359068; doi:10.1186/s13036-021-00270-9)
Supplement: Supplementary file 8 — Additional file 8. [file 13036_2021_270_MOESM8_ESM.docx]

| **Table S1: Primers for Testing and Sequencing** | | | |
| --- | --- | --- | --- |
| **Primer** | **Sequences (5'to3')** | **Base Number** | **Note** |
| Primer Seq-F | gctcttacttcttcgcctctgc | 22 |  |
| Primer Seq-R | tacccacgccgcggttattg | 20 |  |
| Primer Test-F | actggagagattcttcgcaagc | 22 |  |
