# Supplementary material for: CRISPR/Cas9 mediated T7 RNA polymerase gene knock-in in E. coli BW25113 makes T7 expression system work efficiently
Source: J Biol Eng. 2021 Aug 12;15:22. doi: 10.1186/s13036-021-00270-9 (PMC8359068; doi:10.1186/s13036-021-00270-9)
Supplement: Supplementary file 9 — Additional file 9. [file 13036_2021_270_MOESM9_ESM.docx]

| **Table S2: Primers for Construction of T7 Promoter Variants Library** | | | |
| --- | --- | --- | --- |
| **Primer** | **Sequences (5'to3')** | **Base Number** | **Note** |
| Primer T7-R | atttcctaatgcaggagtcgcata | 24 | Embellished by 5’-phosphorylation |
| Primer 5A-F | taatacgactcaAtataggggaattgtgagc | 31 | Positions that are uppercase are variant parts between original T7 and vairant T7. |
| Primer 5T-F | taatacgactcaTtataggggaattgtgagc | 31 |  |
| Primer 5G-F | taatacgactcaGtataggggaattgtgagc | 31 |  |
| Primer 6T-F | taatacgactcTctataggggaattgtgagc | 31 |  |
| Primer 6G-F | taatacgactcGctataggggaattgtgagc | 31 |  |
| Primer 6C-F | taatacgactcCctataggggaattgtgagc | 31 |  |
| Primer 7A-F | taatacgactAactataggggaattgtgagc | 31 |  |
| Primer 7T-F | taatacgactTactataggggaattgtgagc | 31 |  |
| Primer 7G-F | taatacgactGactataggggaattgtgagc | 31 |  |
| Primer 8A-F | taatacgacAcactataggggaattgtgagc | 31 |  |
| Primer 8C-F | taatacgacCcactataggggaattgtgagc | 31 |  |
| Primer 8G-F | taatacgacGcactataggggaattgtgagc | 31 |  |
| Primer 9A-F | taatacgaAtcactataggggaattgtgagc | 31 |  |
| Primer 9T-F | taatacgaTtcactataggggaattgtgagc | 31 |  |
| Primer 9G-F | taatacgaGtcactataggggaattgtgagc | 31 |  |
| Primer 10T-F | taatacgTctcactataggggaattgtgagc | 31 |  |
| Primer 10C-F | taatacgCctcactataggggaattgtgagc | 31 |  |
| Primer 10G-F | taatacgGctcactataggggaattgtgagc | 31 |  |
| Primer 11A-F | taatacAactcactataggggaattgtgagc | 31 |  |
| Primer 11T-F | taatacTactcactataggggaattgtgagc | 31 |  |
| Primer 11C-F | taatacCactcactataggggaattgtgagc | 31 |  |
| Primer 12A-F | taataAgactcactataggggaattgtgagc | 31 |  |
| Primer 12T-F | taataTgactcactataggggaattgtgagc | 31 |  |
| Primer 12G-F | taataGgactcactataggggaattgtgagc | 31 |  |
| Primer 13T-F | taatTcgactcactataggggaattgtgagc | 31 |  |
| Primer 13C-F | taatCcgactcactataggggaattgtgagc | 31 |  |
| Primer 13G-F | taatGcgactcactataggggaattgtgagc | 31 |  |
| Primer 14A-F | taaAacgactcactataggggaattgtgagc | 31 |  |
| Primer 14C-F | taaCacgactcactataggggaattgtgagc | 31 |  |
| Primer 14G-F | taaGacgactcactataggggaattgtgagc | 31 |  |
| Primer 15T-F | taTtacgactcactataggggaattgtgagc | 31 |  |
| Primer 15C-F | taCtacgactcactataggggaattgtgagc | 31 |  |
| Primer 15G-F | taGtacgactcactataggggaattgtgagc | 31 |  |
| Primer 16T-F | tTatacgactcactataggggaattgtgagc | 31 |  |
| Primer 16C-F | tCatacgactcactataggggaattgtgagc | 31 |  |
| Primer 16G-F | tGatacgactcactataggggaattgtgagc | 31 |  |
| Primer 17A-F | Aaatacgactcactataggggaattgtgagc | 31 |  |
| Primer 17C-F | Caatacgactcactataggggaattgtgagc | 31 |  |
| Primer 17G-F | Gaatacgactcactataggggaattgtgagc | 31 |  |
